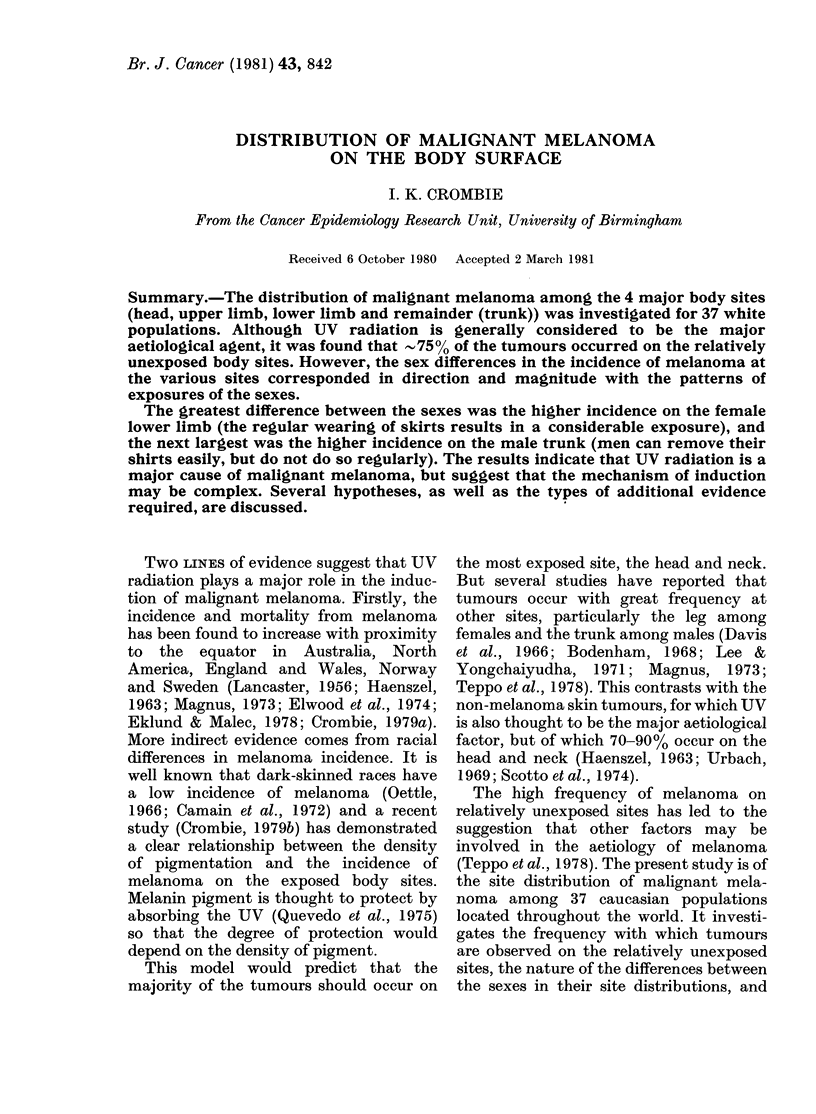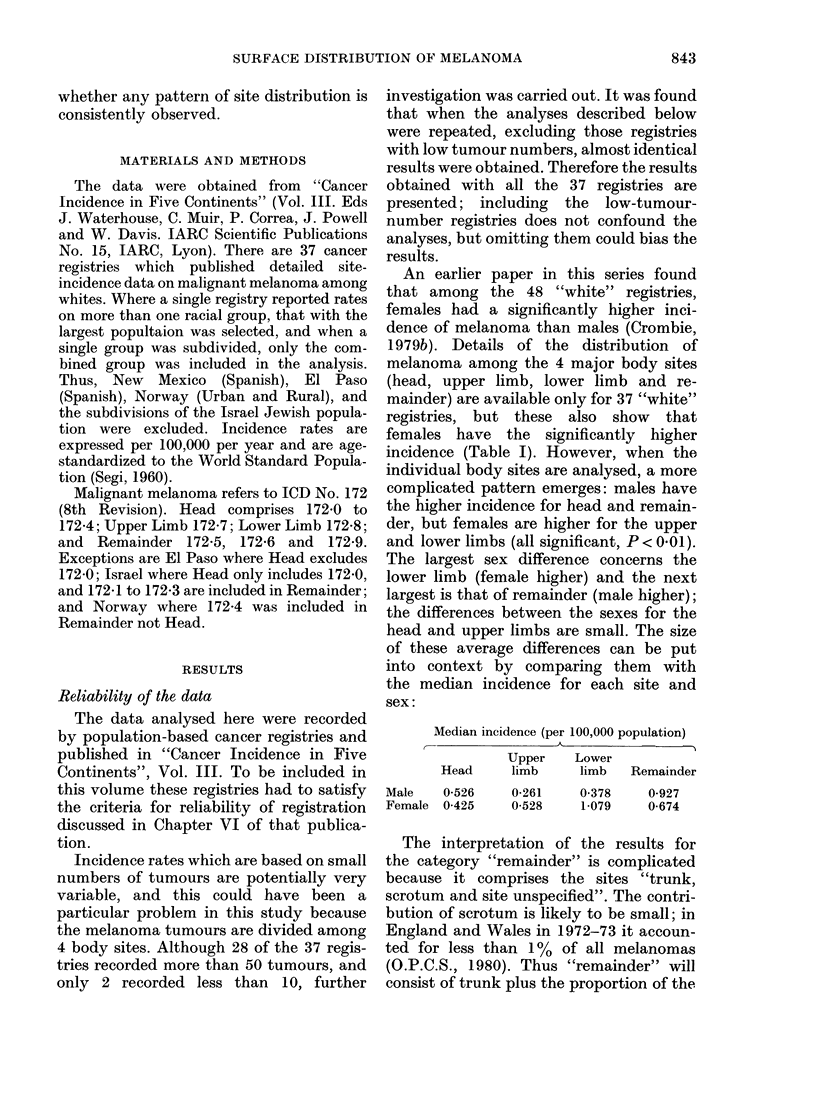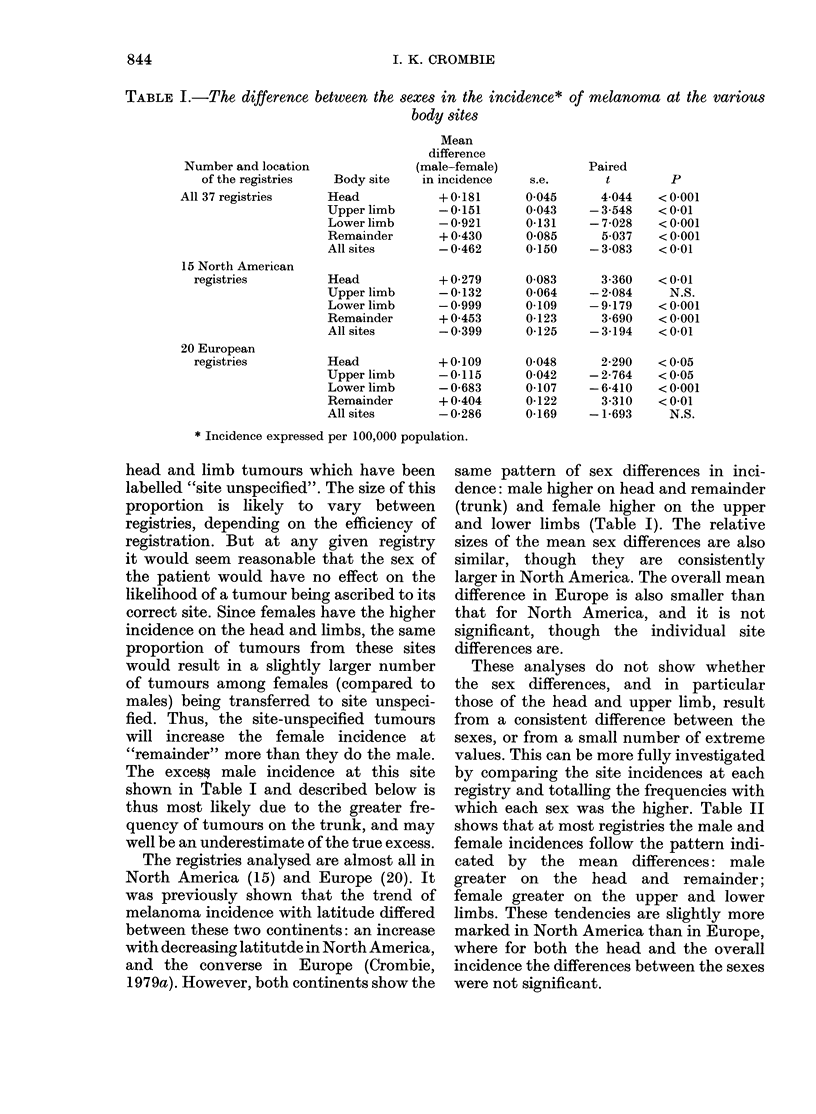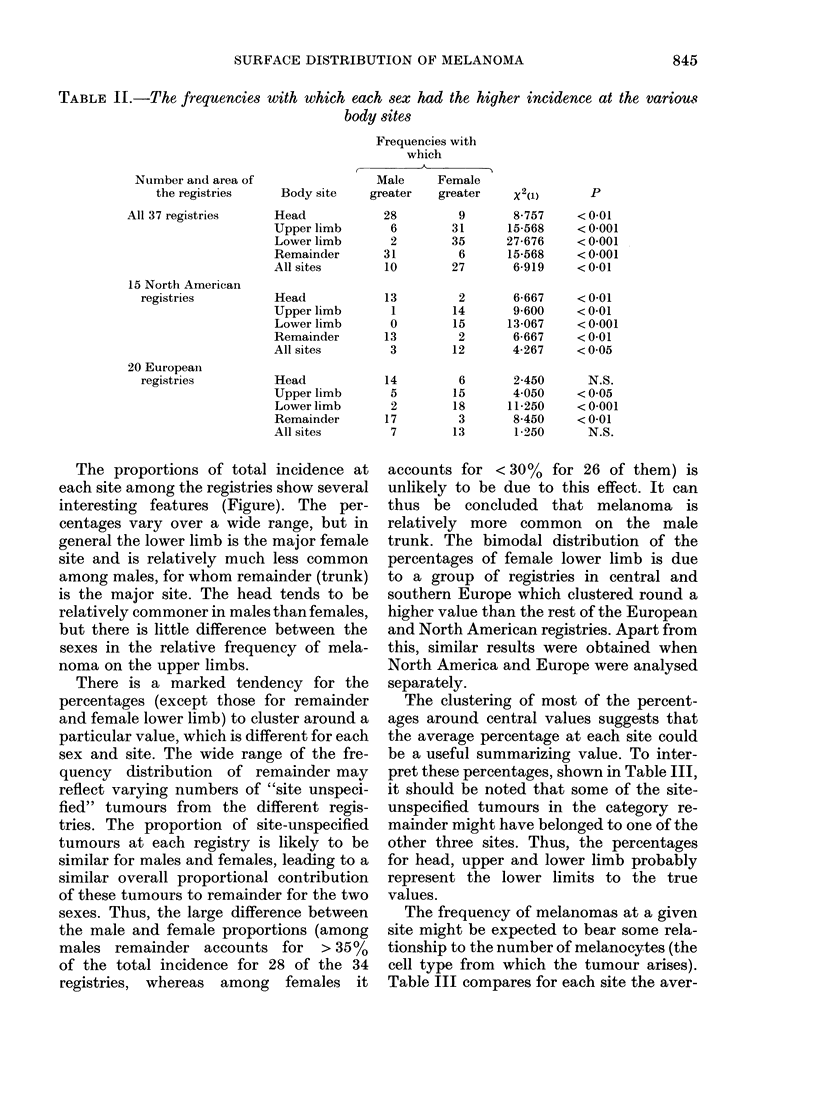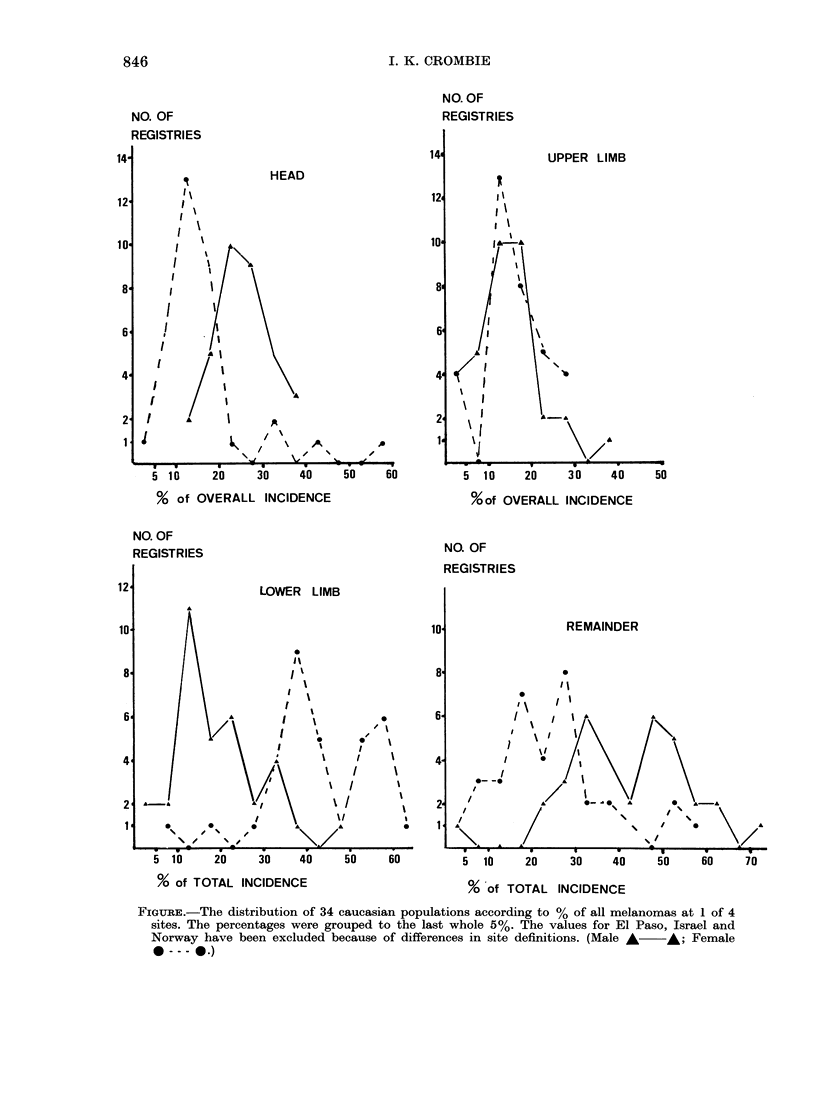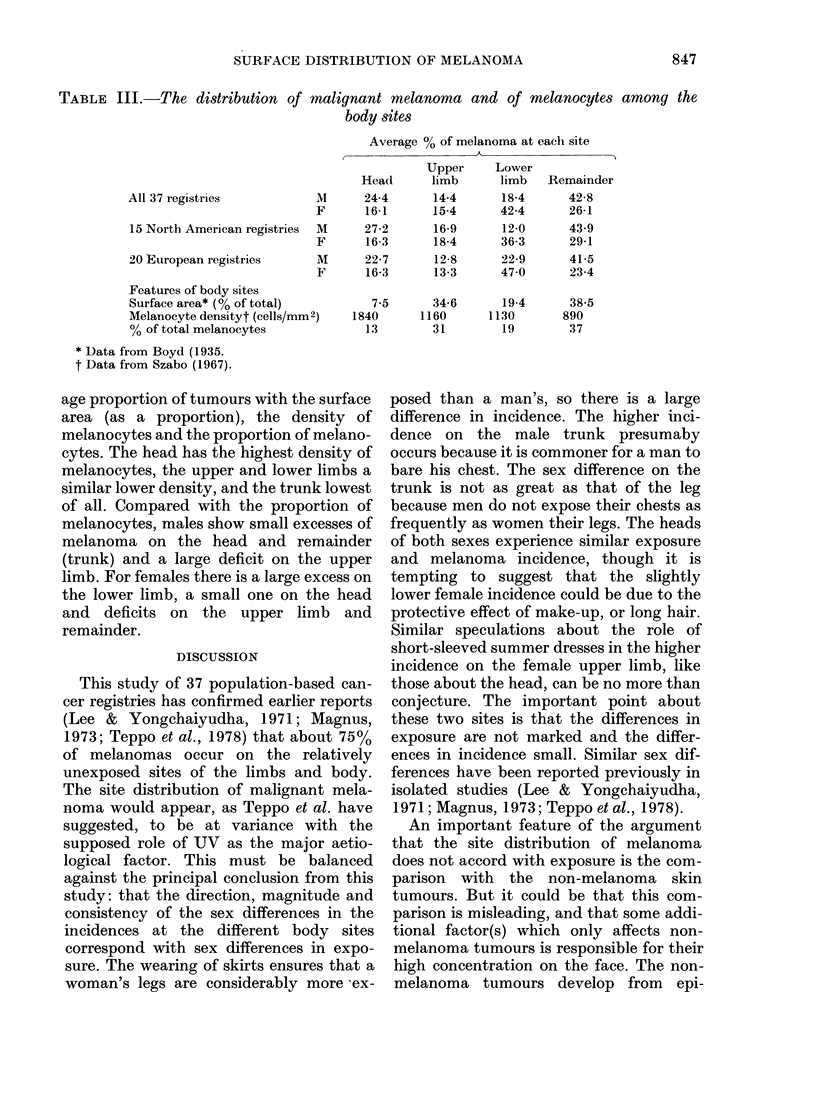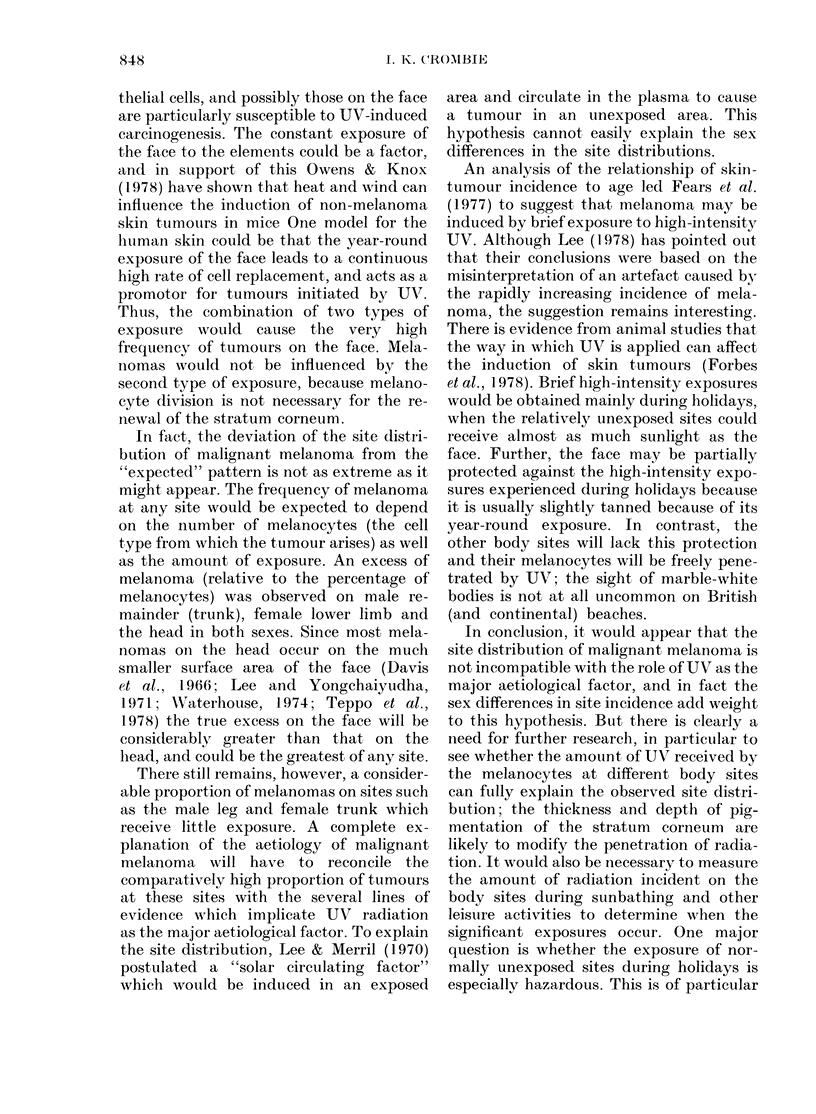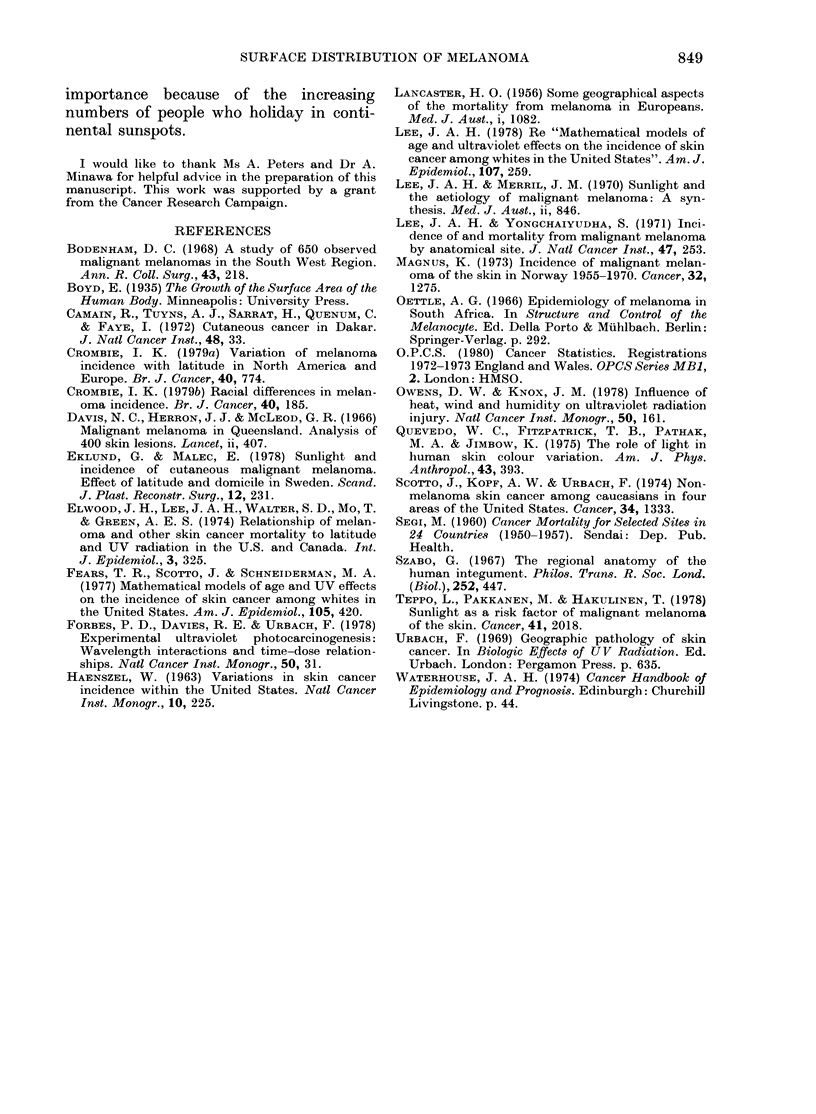# Distribution of malignant melanoma on the body surface.

**DOI:** 10.1038/bjc.1981.123

**Published:** 1981-06

**Authors:** I. K. Crombie

## Abstract

The distribution of malignant melanoma among the 4 major body sites (head, upper limb, lower limb and remainder (trunk) was investigated for 37 white populations. Although UV radiation is generally considered to be the major aetiological agent, it was found that approximately 75% of the tumours occurred on the relatively unexposed body sites. However, the sex differences in the incidence of melanoma at the various sites corresponded in direction and magnitude with the patterns of exposures of the sexes. The greatest difference between the sexes was the higher incidence on the female lower limb (the regular wearing of skirts results in a considerable exposure), and the next largest was the higher incidence on the male trunk (men can remove their shirts easily, but do not do so regularly). The results indicate that UV radiation is a major cause of malignant melanoma, but suggest that the mechanism of induction may be complex. Several hypotheses, as well as the types of additional evidence required, are discussed.


*Br. J. Cancer* (1981) **43**, 842

### DISTRIBUTION OF MALIGNANT MELANOMA ON THE BODY SURFACE

I. K. CROMBIE

*From the Cancer Epidemiology Research Unit, University of Birmingham*

Received 6 October 1980 Accepted 2 March 1981

**Summary.—The distribution of malignant melanoma among the 4 major body sites (head, upper limb, lower limb and remainder (trunk)) was investigated for 37 white populations. Although UV radiation is generally considered to be the major aetiological agent, it was found that ~75% of the tumours occurred on the relatively unexposed body sites. However, the sex differences in the incidence of melanoma at the various sites corresponded in direction and magnitude with the patterns of exposures of the sexes.**

**The greatest difference between the sexes was the higher incidence on the female lower limb (the regular wearing of skirts results in a considerable exposure), and the next largest was the higher incidence on the male trunk (men can remove their shirts easily, but do not do so regularly). The results indicate that UV radiation is a major cause of malignant melanoma, but suggest that the mechanism of induction may be complex. Several hypotheses, as well as the types of additional evidence required, are discussed.**

Two LINES of evidence suggest that UV radiation plays a major role in the induction of malignant melanoma. Firstly, the incidence and mortality from melanoma has been found to increase with proximity to the equator in Australia, North America, England and Wales, Norway and Sweden (Lancaster, 1956; Haenszel, 1963; Magnus, 1973; Elwood *et al.*, 1974; Eklund & Malec, 1978; Crombie, 1979*a*). More indirect evidence comes from racial differences in melanoma incidence. It is well known that dark-skinned races have a low incidence of melanoma (Oettle, 1966; Camain *et al.*, 1972) and a recent study (Crombie, 1979*b*) has demonstrated a clear relationship between the density of pigmentation and the incidence of melanoma on the exposed body sites. Melanin pigment is thought to protect by absorbing the UV (Quevedo *et al.*, 1975) so that the degree of protection would depend on the density of pigment.

This model would predict that the majority of the tumours should occur on the most exposed site, the head and neck. But several studies have reported that tumours occur with great frequency at other sites, particularly the leg among females and the trunk among males (Davis *et al.*, 1966; Bodenham, 1968; Lee & Yongchaiyudha, 1971; Magnus, 1973; Teppo *et al.*, 1978). This contrasts with the non-melanoma skin tumours, for which UV is also thought to be the major aetiological factor, but of which 70–90% occur on the head and neck (Haenszel, 1963; Urbach, 1969; Scotto *et al.*, 1974).

The high frequency of melanoma on relatively unexposed sites has led to the suggestion that other factors may be involved in the aetiology of melanoma (Teppo *et al.*, 1978). The present study is of the site distribution of malignant melanoma among 37 caucasian populations located throughout the world. It investigates the frequency with which tumours are observed on the relatively unexposed sites, the nature of the differences between the sexes in their site distributions, and whether any pattern of site distribution is consistently observed.

### MATERIALS AND METHODS

The data were obtained from "Cancer Incidence in Five Continents" (Vol. III. Eds J. Waterhouse, C. Muir, P. Correa, J. Powell and W. Davis. IARC Scientific Publications No. 15, IARC, Lyon). There are 37 cancer registries which published detailed site-incidence data on malignant melanoma among whites. Where a single registry reported rates on more than one racial group, that with the largest popultaion was selected, and when a single group was subdivided, only the combined group was included in the analysis. Thus, New Mexico (Spanish), El Paso (Spanish), Norway (Urban and Rural), and the subdivisions of the Israel Jewish population were excluded. Incidence rates are expressed per 100,000 per year and are age-standardized to the World Standard Population (Segi, 1960).

Malignant melanoma refers to ICD No. 172 (8th Revision). Head comprises 172·0 to 172·4; Upper Limb 172·7; Lower Limb 172·8; and Remainder 172·5, 172·6 and 172·9. Exceptions are El Paso where Head excludes 172·0; Israel where Head only includes 172·0, and 172·1 to 172·3 are included in Remainder; and Norway where 172·4 was included in Remainder not Head.

### RESULTS

### *Reliability of the data*

The data analysed here were recorded by population-based cancer registries and published in "Cancer Incidence in Five Continents", Vol. III. To be included in this volume these registries had to satisfy the criteria for reliability of registration discussed in Chapter VI of that publication.

Incidence rates which are based on small numbers of tumours are potentially very variable, and this could have been a particular problem in this study because the melanoma tumours are divided among 4 body sites. Although 28 of the 37 registries recorded more than 50 tumours, and only 2 recorded less than 10, further investigation was carried out. It was found that when the analyses described below were repeated, excluding those registries with low tumour numbers, almost identical results were obtained. Therefore the results obtained with all the 37 registries are presented; including the low-tumour-number registries does not confound the analyses, but omitting them could bias the results.

An earlier paper in this series found that among the 48 "white" registries, females had a significantly higher incidence of melanoma than males (Crombie, 1979*b*). Details of the distribution of melanoma among the 4 major body sites (head, upper limb, lower limb and remainder) are available only for 37 "white" registries, but these also show that females have the significantly higher incidence (Table I). However, when the individual body sites are analysed, a more complicated pattern emerges: males have the higher incidence for head and remainder, but females are higher for the upper and lower limbs (all significant, $P < 0{\cdot}01$). The largest sex difference concerns the lower limb (female higher) and the next largest is that of remainder (male higher); the differences between the sexes for the head and upper limbs are small. The size of these average differences can be put into context by comparing them with the median incidence for each site and sex:

| | Median incidence (per 100,000 population) | | | |
|---|---|---|---|---|
| | Head | Upper limb | Lower limb | Remainder |
| Male | 0·526 | 0·261 | 0·378 | 0·927 |
| Female | 0·425 | 0·528 | 1·079 | 0·674 |

The interpretation of the results for the category "remainder" is complicated because it comprises the sites "trunk, scrotum and site unspecified". The contribution of scrotum is likely to be small; in England and Wales in 1972–73 it accounted for less than 1% of all melanomas (O.P.C.S., 1980). Thus "remainder" will consist of trunk plus the proportion of the

TABLE I.—*The difference between the sexes in the incidence* of melanoma at the various body sites*

| Number and location of the registries | Body site | Mean difference (male–female) in incidence | s.e. | Paired $t$ | $P$ |
|---|---|---|---|---|---|
| All 37 registries | Head | +0·181 | 0·045 | 4·044 | <0·001 |
| | Upper limb | −0·151 | 0·043 | −3·548 | <0·01 |
| | Lower limb | −0·921 | 0·131 | −7·028 | <0·001 |
| | Remainder | +0·430 | 0·085 | 5·037 | <0·001 |
| | All sites | −0·462 | 0·150 | −3·083 | <0·01 |
| 15 North American registries | Head | +0·279 | 0·083 | 3·360 | <0·01 |
| | Upper limb | −0·132 | 0·064 | −2·084 | N.S. |
| | Lower limb | −0·999 | 0·109 | −9·179 | <0·001 |
| | Remainder | +0·453 | 0·123 | 3·690 | <0·001 |
| | All sites | −0·399 | 0·125 | −3·194 | <0·01 |
| 20 European registries | Head | +0·109 | 0·048 | 2·290 | <0·05 |
| | Upper limb | −0·115 | 0·042 | −2·764 | <0·05 |
| | Lower limb | −0·683 | 0·107 | −6·410 | <0·001 |
| | Remainder | +0·404 | 0·122 | 3·310 | <0·01 |
| | All sites | −0·286 | 0·169 | −1·693 | N.S. |

* Incidence expressed per 100,000 population.

head and limb tumours which have been labelled "site unspecified". The size of this proportion is likely to vary between registries, depending on the efficiency of registration. But at any given registry it would seem reasonable that the sex of the patient would have no effect on the likelihood of a tumour being ascribed to its correct site. Since females have the higher incidence on the head and limbs, the same proportion of tumours from these sites would result in a slightly larger number of tumours among females (compared to males) being transferred to site unspecified. Thus, the site-unspecified tumours will increase the female incidence at "remainder" more than they do the male. The excess male incidence at this site shown in Table I and described below is thus most likely due to the greater frequency of tumours on the trunk, and may well be an underestimate of the true excess.

The registries analysed are almost all in North America (15) and Europe (20). It was previously shown that the trend of melanoma incidence with latitude differed between these two continents: an increase with decreasing latitutde in North America, and the converse in Europe (Crombie, 1979*a*). However, both continents show the same pattern of sex differences in incidence: male higher on head and remainder (trunk) and female higher on the upper and lower limbs (Table I). The relative sizes of the mean sex differences are also similar, though they are consistently larger in North America. The overall mean difference in Europe is also smaller than that for North America, and it is not significant, though the individual site differences are.

These analyses do not show whether the sex differences, and in particular those of the head and upper limb, result from a consistent difference between the sexes, or from a small number of extreme values. This can be more fully investigated by comparing the site incidences at each registry and totalling the frequencies with which each sex was the higher. Table II shows that at most registries the male and female incidences follow the pattern indicated by the mean differences: male greater on the head and remainder; female greater on the upper and lower limbs. These tendencies are slightly more marked in North America than in Europe, where for both the head and the overall incidence the differences between the sexes were not significant.

TABLE II.—*The frequencies with which each sex had the higher incidence at the various body sites*

| Number and area of the registries | Body site | Frequencies with which: Male greater | Female greater | $\chi^2_{(1)}$ | $P$ |
|---|---|---|---|---|---|
| All 37 registries | Head | 28 | 9 | 8·757 | <0·01 |
| | Upper limb | 6 | 31 | 15·568 | <0·001 |
| | Lower limb | 2 | 35 | 27·676 | <0·001 |
| | Remainder | 31 | 6 | 15·568 | <0·001 |
| | All sites | 10 | 27 | 6·919 | <0·01 |
| 15 North American registries | Head | 13 | 2 | 6·667 | <0·01 |
| | Upper limb | 1 | 14 | 9·600 | <0·01 |
| | Lower limb | 0 | 15 | 13·067 | <0·001 |
| | Remainder | 13 | 2 | 6·667 | <0·01 |
| | All sites | 3 | 12 | 4·267 | <0·05 |
| 20 European registries | Head | 14 | 6 | 2·450 | N.S. |
| | Upper limb | 5 | 15 | 4·050 | <0·05 |
| | Lower limb | 2 | 18 | 11·250 | <0·001 |
| | Remainder | 17 | 3 | 8·450 | <0·01 |
| | All sites | 7 | 13 | 1·250 | N.S. |

The proportions of total incidence at each site among the registries show several interesting features (Figure). The percentages vary over a wide range, but in general the lower limb is the major female site and is relatively much less common among males, for whom remainder (trunk) is the major site. The head tends to be relatively commoner in males than females, but there is little difference between the sexes in the relative frequency of melanoma on the upper limbs.

There is a marked tendency for the percentages (except those for remainder and female lower limb) to cluster around a particular value, which is different for each sex and site. The wide range of the frequency distribution of remainder may reflect varying numbers of "site unspecified" tumours from the different registries. The proportion of site-unspecified tumours at each registry is likely to be similar for males and females, leading to a similar overall proportional contribution of these tumours to remainder for the two sexes. Thus, the large difference between the male and female proportions (among males remainder accounts for >35% of the total incidence for 28 of the 34 registries, whereas among females it accounts for <30% for 26 of them) is unlikely to be due to this effect. It can thus be concluded that melanoma is relatively more common on the male trunk. The bimodal distribution of the percentages of female lower limb is due to a group of registries in central and southern Europe which clustered round a higher value than the rest of the European and North American registries. Apart from this, similar results were obtained when North America and Europe were analysed separately.

The clustering of most of the percentages around central values suggests that the average percentage at each site could be a useful summarizing value. To interpret these percentages, shown in Table III, it should be noted that some of the site-unspecified tumours in the category remainder might have belonged to one of the other three sites. Thus, the percentages for head, upper and lower limb probably represent the lower limits to the true values.

The frequency of melanomas at a given site might be expected to bear some relationship to the number of melanocytes (the cell type from which the tumour arises). Table III compares for each site the aver-

FIGURE.—The distribution of 34 caucasian populations according to % of all melanomas at 1 of 4 sites. The percentages were grouped to the last whole 5%. The values for El Paso, Israel and Norway have been excluded because of differences in site definitions. (Male ▲——▲; Female ●- - - ●.)

TABLE III.—*The distribution of malignant melanoma and of melanocytes among the body sites*

| | | Average % of melanoma at each site | | | |
|---|---|---|---|---|---|
| | | Head | Upper limb | Lower limb | Remainder |
| All 37 registries | M | 24·4 | 14·4 | 18·4 | 42·8 |
| | F | 16·1 | 15·4 | 42·4 | 26·1 |
| 15 North American registries | M | 27·2 | 16·9 | 12·0 | 43·9 |
| | F | 16·3 | 18·4 | 36·3 | 29·1 |
| 20 European registries | M | 22·7 | 12·8 | 22·9 | 41·5 |
| | F | 16·3 | 13·3 | 47·0 | 23·4 |
| Features of body sites | | | | | |
| Surface area* (% of total) | | 7·5 | 34·6 | 19·4 | 38·5 |
| Melanocyte density† (cells/mm$^2$) | | 1840 | 1160 | 1130 | 890 |
| % of total melanocytes | | 13 | 31 | 19 | 37 |

* Data from Boyd (1935.

† Data from Szabo (1967).

age proportion of tumours with the surface area (as a proportion), the density of melanocytes and the proportion of melanocytes. The head has the highest density of melanocytes, the upper and lower limbs a similar lower density, and the trunk lowest of all. Compared with the proportion of melanocytes, males show small excesses of melanoma on the head and remainder (trunk) and a large deficit on the upper limb. For females there is a large excess on the lower limb, a small one on the head and deficits on the upper limb and remainder.

### DISCUSSION

This study of 37 population-based cancer registries has confirmed earlier reports (Lee & Yongchaiyudha, 1971; Magnus, 1973; Teppo *et al.*, 1978) that about 75% of melanomas occur on the relatively unexposed sites of the limbs and body. The site distribution of malignant melanoma would appear, as Teppo *et al.* have suggested, to be at variance with the supposed role of UV as the major aetiological factor. This must be balanced against the principal conclusion from this study: that the direction, magnitude and consistency of the sex differences in the incidences at the different body sites correspond with sex differences in exposure. The wearing of skirts ensures that a woman's legs are considerably more exposed than a man's, so there is a large difference in incidence. The higher incidence on the male trunk presumaby occurs because it is commoner for a man to bare his chest. The sex difference on the trunk is not as great as that of the leg because men do not expose their chests as frequently as women their legs. The heads of both sexes experience similar exposure and melanoma incidence, though it is tempting to suggest that the slightly lower female incidence could be due to the protective effect of make-up, or long hair. Similar speculations about the role of short-sleeved summer dresses in the higher incidence on the female upper limb, like those about the head, can be no more than conjecture. The important point about these two sites is that the differences in exposure are not marked and the differences in incidence small. Similar sex differences have been reported previously in isolated studies (Lee & Yongchaiyudha, 1971; Magnus, 1973; Teppo *et al.*, 1978).

An important feature of the argument that the site distribution of melanoma does not accord with exposure is the comparison with the non-melanoma skin tumours. But it could be that this comparison is misleading, and that some additional factor(s) which only affects non-melanoma tumours is responsible for their high concentration on the face. The non-melanoma tumours develop from epithelial cells, and possibly those on the face are particularly susceptible to UV-induced carcinogenesis. The constant exposure of the face to the elements could be a factor, and in support of this Owens & Knox (1978) have shown that heat and wind can influence the induction of non-melanoma skin tumours in mice One model for the human skin could be that the year-round exposure of the face leads to a continuous high rate of cell replacement, and acts as a promotor for tumours initiated by UV. Thus, the combination of two types of exposure would cause the very high frequency of tumours on the face. Melanomas would not be influenced by the second type of exposure, because melanocyte division is not necessary for the renewal of the stratum corneum.

In fact, the deviation of the site distribution of malignant melanoma from the "expected" pattern is not as extreme as it might appear. The frequency of melanoma at any site would be expected to depend on the number of melanocytes (the cell type from which the tumour arises) as well as the amount of exposure. An excess of melanoma (relative to the percentage of melanocytes) was observed on male remainder (trunk), female lower limb and the head in both sexes. Since most melanomas on the head occur on the much smaller surface area of the face (Davis *et al.*, 1966; Lee and Yongchaiyudha, 1971; Waterhouse, 1974; Teppo *et al.*, 1978) the true excess on the face will be considerably greater than that on the head, and could be the greatest of any site.

There still remains, however, a considerable proportion of melanomas on sites such as the male leg and female trunk which receive little exposure. A complete explanation of the aetiology of malignant melanoma will have to reconcile the comparatively high proportion of tumours at these sites with the several lines of evidence which implicate UV radiation as the major aetiological factor. To explain the site distribution, Lee & Merril (1970) postulated a "solar circulating factor" which would be induced in an exposed area and circulate in the plasma to cause a tumour in an unexposed area. This hypothesis cannot easily explain the sex differences in the site distributions.

An analysis of the relationship of skin-tumour incidence to age led Fears *et al.* (1977) to suggest that melanoma may be induced by brief exposure to high-intensity UV. Although Lee (1978) has pointed out that their conclusions were based on the misinterpretation of an artefact caused by the rapidly increasing incidence of melanoma, the suggestion remains interesting. There is evidence from animal studies that the way in which UV is applied can affect the induction of skin tumours (Forbes *et al.*, 1978). Brief high-intensity exposures would be obtained mainly during holidays, when the relatively unexposed sites could receive almost as much sunlight as the face. Further, the face may be partially protected against the high-intensity exposures experienced during holidays because it is usually slightly tanned because of its year-round exposure. In contrast, the other body sites will lack this protection and their melanocytes will be freely penetrated by UV; the sight of marble-white bodies is not at all uncommon on British (and continental) beaches.

In conclusion, it would appear that the site distribution of malignant melanoma is not incompatible with the role of UV as the major aetiological factor, and in fact the sex differences in site incidence add weight to this hypothesis. But there is clearly a need for further research, in particular to see whether the amount of UV received by the melanocytes at different body sites can fully explain the observed site distribution; the thickness and depth of pigmentation of the stratum corneum are likely to modify the penetration of radiation. It would also be necessary to measure the amount of radiation incident on the body sites during sunbathing and other leisure activities to determine when the significant exposures occur. One major question is whether the exposure of normally unexposed sites during holidays is especially hazardous. This is of particular importance because of the increasing numbers of people who holiday in continental sunspots.

I would like to thank Ms A. Peters and Dr A. Minawa for helpful advice in the preparation of this manuscript. This work was supported by a grant from the Cancer Research Campaign.

### REFERENCES

BODENHAM, D. C. (1968) A study of 650 observed malignant melanomas in the South West Region. *Ann. R. Coll. Surg.*, **43**, 218.

BOYD, E. (1935) *The Growth of the Surface Area of the Human Body*. Minneapolis: University Press.

CAMAIN, R., TUYNS, A. J., SARRAT, H., QUENUM, C. & FAYE, I. (1972) Cutaneous cancer in Dakar. *J. Natl Cancer Inst.*, **48**, 33.

CROMBIE, I. K. (1979*a*) Variation of melanoma incidence with latitude in North America and Europe. *Br. J. Cancer*, **40**, 774.

CROMBIE, I. K. (1979*b*) Racial differences in melanoma incidence. *Br. J. Cancer*, **40**, 185.

DAVIS, N. C., HERRON, J. J. & MCLEOD, G. R. (1966) Malignant melanoma in Queensland. Analysis of 400 skin lesions. *Lancet*, ii, 407.

EKLUND, G. & MALEC, E. (1978) Sunlight and incidence of cutaneous malignant melanoma. Effect of latitude and domicile in Sweden. *Scand. J. Plast. Reconstr. Surg.*, **12**, 231.

ELWOOD, J. H., LEE, J. A. H., WALTER, S. D., MO, T. & GREEN, A. E. S. (1974) Relationship of melanoma and other skin cancer mortality to latitude and UV radiation in the U.S. and Canada. *Int. J. Epidemiol.*, **3**, 325.

FEARS, T. R., SCOTTO, J. & SCHNEIDERMAN, M. A. (1977) Mathematical models of age and UV effects on the incidence of skin cancer among whites in the United States. *Am. J. Epidemiol.*, **105**, 420.

FORBES, P. D., DAVIES, R. E. & URBACH, F. (1978) Experimental ultraviolet photocarcinogenesis: Wavelength interactions and time-dose relationships. *Natl Cancer Inst. Monogr.*, **50**, 31.

HAENSZEL, W. (1963) Variations in skin cancer incidence within the United States. *Natl Cancer Inst. Monogr.*, **10**, 225.

LANCASTER, H. O. (1956) Some geographical aspects of the mortality from melanoma in Europeans. *Med. J. Aust.*, i, 1082.

LEE, J. A. H. (1978) Re "Mathematical models of age and ultraviolet effects on the incidence of skin cancer among whites in the United States". *Am. J. Epidemiol.*, **107**, 259.

LEE, J. A. H. & MERRIL, J. M. (1970) Sunlight and the aetiology of malignant melanoma: A synthesis. *Med. J. Aust.*, ii, 846.

LEE, J. A. H. & YONGCHAIYUDHA, S. (1971) Incidence of and mortality from malignant melanoma by anatomical site. *J. Natl Cancer Inst.*, **47**, 253.

MAGNUS, K. (1973) Incidence of malignant melanoma of the skin in Norway 1955–1970. *Cancer*, **32**, 1275.

OETTLE, A. G. (1966) Epidemiology of melanoma in South Africa. In *Structure and Control of the Melanocyte*. Ed. Della Porto & Mühlbach. Berlin: Springer-Verlag. p. 292.

O.P.C.S. (1980) Cancer Statistics. Registrations 1972–1973 England and Wales. *OPCS Series MB1*, **2**. London: HMSO.

OWENS, D. W. & KNOX, J. M. (1978) Influence of heat, wind and humidity on ultraviolet radiation injury. *Natl Cancer Inst. Monogr.*, **50**, 161.

QUEVEDO, W. C., FITZPATRICK, T. B., PATHAK, M. A. & JIMBOW, K. (1975) The role of light in human skin colour variation. *Am. J. Phys. Anthropol.*, **43**, 393.

SCOTTO, J., KOPF, A. W. & URBACH, F. (1974) Non-melanoma skin cancer among caucasians in four areas of the United States. *Cancer*, **34**, 1333.

SEGI, M. (1960) *Cancer Mortality for Selected Sites in 24 Countries* (1950–1957). Sendai: Dep. Pub. Health.

SZABO, G. (1967) The regional anatomy of the human integument. *Philos. Trans. R. Soc. Lond.* (*Biol.*), **252**, 447.

TEPPO, L., PAKKANEN, M. & HAKULINEN, T. (1978) Sunlight as a risk factor of malignant melanoma of the skin. *Cancer*, **41**, 2018.

URBACH, F. (1969) Geographic pathology of skin cancer. In *Biologic Effects of UV Radiation*. Ed. Urbach. London: Pergamon Press. p. 635.

WATERHOUSE, J. A. H. (1974) *Cancer Handbook of Epidemiology and Prognosis*. Edinburgh: Churchill Livingstone. p. 44.